# OLDER CANCER SURVIVORS’ POST-TRAUMATIC GROWTH DURING THE COVID-19 PANDEMIC

**DOI:** 10.1093/geroni/igac059.2838

**Published:** 2022-12-20

**Authors:** Kaleigh Ligus, Emily Fritzson, Caroline Salafia, Crystal Park, Keith Bellizzi

**Affiliations:** University of Connecticut, Storrs, Connecticut, United States; University of Connecticut, Storrs, Connecticut, United States; University of Connecticut, Storrs, Connecticut, United States; University of Connecticut, Storrs, Connecticut, United States; University of Connecticut, Storrs, Connecticut, United States

## Abstract

Older adults and immune-compromised individuals (e.g., cancer survivors) were quickly identified as high-risk groups during the COVID-19 pandemic. Research has examined adverse psychological consequences due to the pandemic in older adults with chronic conditions. However, no research has examined the potential for positive consequences of COVID-19 in older adults with cancer. The current study examined the association between COVID-19 risk, stress, exposure, and perceived post-traumatic growth (PPTG) in adults with cancer. Cancer survivors (n = 231) enrolled in an ongoing longitudinal study completed a one-time COVID-19 experience assessment, including PPTG as a result of the COVID pandemic. Participants (mean age = 68 years) had completed primary treatment for breast, prostate, or colorectal cancer within the past two years. On average, survivors reported modest PPTG related to COVID-19 (mean = 6.03, range = 0–20). Using linear regression, we found that gender, cancer type, and marital status were related to PPTG. Higher COVID-19 risk, more COVID-19 stressors, and higher illness exposure (e.g., having had COVID-19) were significantly positively associated with PPTG scores (ps < .05). In spite of the potential adverse psychological consequences of the COVID-19 pandemic, these results suggest adults with cancer, in our sample, report modest PPTG. Higher COVID-19-related perceived risk, stress, and exposure predicted higher PPTG scores. These findings are interesting in light of studies that suggest PPTG occurs only after time passes from the stressor. Future research could examine how coping with cancer may influence coping with a co-existing or subsequent stressor and its relationship to PPTG.

